# Actinomycetes Diversity among rRNA Gene Clones and Cellular Isolates from Sambhar Salt Lake, India

**DOI:** 10.1155/2013/781301

**Published:** 2013-11-06

**Authors:** A. K. Yadav, S. Vardhan, S. Kashyap, M. Yandigeri, D. K. Arora

**Affiliations:** ^1^National Bureau of Agriculturally Important Microorganisms, Kushmaur, Katholi, Maunath Bhanjan, Uttar Pradesh 275101, India; ^2^National Bureau of Agriculturally Important Insects, H.A. Farm Post, Bellary Road, Bangalore, Karnatka 560024, India; ^3^Sher-e-Kasmir University of Agriculture Science & Technology of Jammu, Main Campus, Chatta, Jammu (J&K) 180009, India

## Abstract

The vertical stratification of actinomycetes diversity in Sambhar salt lake (India's largest salt lake) was investigated by using cultivable and uncultivable approaches. The isolates from cultured approaches were clustered on the basis of cultural, morphological, biochemical, and cell wall characteristics, and results were further strengthened by 16S rDNA-RFLP into five major groups. 16S rDNA sequencing of the representative isolates from each clusters was identified as belonging to *Streptomyces, Actinopolyspora, Microbispora, Saccharopolyspora*, and *Actinoplanes* genera, while culture independent group was established as *Streptomyces* (130 clones, 20 OTUs), *Micromonospora* (96 clones, 7 OTUs), *Streptosporangium* (79 clones, 9 OTUs), *Thermomonospora* (46 clones, 8 OTUs), and *Dactylosporangium* (58 clones, 8 OTUs). The diversity assessment using Shannon and Wiener index was found to be 1.55, 1.52, 1.55, and 1.49 from surface lake water, at depth of 1.5 m, shallow layer of water with algal population, and finally at depth of 2.5 m, respectively. We observed diversity in terms of the species richness as *Streptomyces* is dominant genus in both culture dependent and culture independent techniques followed by *Microbispora * (culture dependent methods) and *Micromonospora* (culture independent method) genera, respectively.

## 1. Introduction

 Aquatic realms of biosphere cover 75% of the earth's surface and represent 95% of the biosphere which provides the largest inhabitable space for living organisms, particularly microbes [1, 2]. The Sambhar salt lake has attracted attention over the past century due to its pronounced hypersalinity and being a key wintering area for northern shoveller and black-headed gull and other northern Asian migratory birds. Earlier, hypothesis for hypersalinity has suggested a wind born source of salt from Ran of Kutch [3], an inland Tethys sea during the Tertiary period [4], and dissolution of halite bed in lake area [5]. More recent isotopic studies have clearly refuted the marine origin and have suggested that the lake brine is yearly replenished by environmental precipitation and surface runoff mediated weathering [6, 7]. In the last two decades, several attempts have been made for phylogenetic characterization of microflora from salt lake in different parts of the world [8]. Though soil actinomycete diversity has been extensively studied, relatively few efforts with aquatic actinomycetes have been attempted. Research on the biodiversity of aquatic actinomycetes is not only important for basic studies but also necessary for its exploitation. The cloning of rRNA genes (rDNAs) from natural ecosystems precipitated a fundamental shift in microbial ecology away from the study of cultured strains and towards molecular approaches that emphasized the importance of *in situ* diversity. The great plate count anomalies [9] led to the formulation of two nonexclusive hypotheses that could be applied generally to many ecosystems: firstly bacterial communities are composed of known species that are capable of forming colonies on agar plates but do so with low efficiency, and secondly it is composed of many unknown species that cannot easily be grown on common microbiological media [10]. There are only few reports from Sambhar salt lake, belonging to green alga *Dunaliella salina* and the bacterium *Serratia sambhariana*. Till date, there are no reports available on the actinomycetes diversity from Sambhar lake. The evaluation of actinomycetes population is required for understanding its biogeography, community assembly, and ecological processes [11] within a particular exotic niche. Exotic niches harbor population of microorganisms that are a source of several commercially important products like enzymes and antibiotics, one such extreme niche the Sambhar salt lake has maintained some degree of pristine quality and their biotechnological potential has remained unrealized. This is the first report to decipher the actinomycetes diversity from Sambhar lake. Recent studies using both cultural independent molecular approaches and culture-based methods demonstrated abundant novel actinobacterial assemblage in aquatic ecosystems; with this perception in this study, we combined both the culture dependent and culture independent methods for the diversity analysis which enables us to access diversity more precisely than does either method alone [12, 13]. In the present work, we reported the results of a study in which *Hha*I/*Cfo*I and *Taq*I restriction fragment length polymorphism (RFLP) patterns and partial gene sequences were used to compare the 16S rDNAs subunit isolates to actinobacterial 16S rDNAs cloned, with the intention of screening these isolates for potentially useful enzymes and metabolites. Here we decipher the diversity of the actinomycetes in Sambhar salt lake.

## 2. Material and Methods 

### 2.1. Site Description, Samples Collection, and Water Analysis

**Figure 1:**
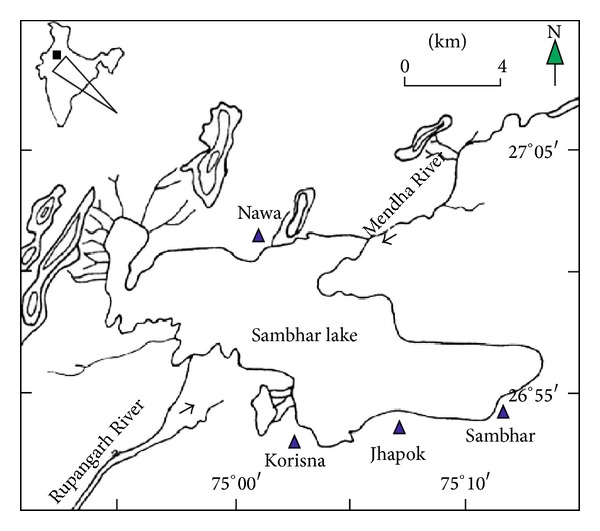
Schematic map of sampling site, Sambhar salt lake, India.

Sambhar is an elliptical shallow lake (26.967°N & 75.083°E), with a maximum length of 22.5 Km and width of 3.2 to 11.2 Km with the total catchment area of about 225 Km^2^ with an average depth of 1–3 m (Figure 1). The lake is bounded by Aravali hill with precipitation of 100–500 mm of South Western monsoon and an average annual temperature of 23°C to a maximum of 45°C. Water samples (0.8–1 L) were collected in a sterile scotch bottles by submerging at surface, 1.5 m, 2.5 m depth of lake in random manner. Samples were brought into laboratory under refrigerated condition for further studies. Before analysis the samples were brought to room temperature and their chemical characteristics were determined using standard methods. The pH was measured by pH meter, chloride was determined by argentometric method, and sulphate was estimated gravimetrically after precipitation in HCl as barium sulphate [14]. Macronutrients (Na, K, Ca, and Mg) were determined using atomic absorption spectrophotometer (Brunswick 301). 

### 2.2. Isolation and Characterization of Actinomycetes Isolates

Isolation of actinomycetes was done by enrichment method of Wang et al. [15]. Standard methods were used to study the morphological, physiological, and biochemical traits [16–19]. Morphological characteristics of actinomycetes were observed on light microscopy (GX10) [16], as well as on scanning electron microscopy [20]. For chemotaxonomic characterization, cell wall preparations and whole cell analysis were done by the modified procedure of Becker et al. [21]. 

### 2.3. 16S rDNA and Clone Library

Genomic DNA of cellular isolates and environmental samples were extracted by the modified method of Boudjella et al. [22] and Fuhrman et al. [23]. PCR amplification of 16S rDNA for cellular isolates was carried out by using universal primers fD1 (5′-GAGTTTGATCCTGGCTCA-3′) and RP2 (5′-CGGCTACCTTGTTACGACTT-3′) Weisburg et al. [24], while environmental sample DNA, genus specific 16S rDNA primers (*Streptomyces*, *Micromonospora*, *Dactylosporangium*, *Thermomonospora*, and *Streptosporangium* genera), and amplification conditions were used according to Monciardini et al. [25]. Analysis of the 16S r RNA genes was conducted according to the method of Hobel et al. [26]. 

### 2.4. Restriction Fragment Lengths Polymorphism Analysis (RFLP) and Sequencing

PCR product from cellular isolates and plasmids from gene clones were restricted with a set of two restriction enzymes separately (*Hha*I/*Cfo*I and *Taq*I) according to the manufacturer's instructions (Genei, India). The banding pattern development was analyzed by horizontal electrophoresis in 2.5% agarose gels and documented on a gel documentation system (Alpha Imager, USA). PCR products were purified using purification kit (Genei, India). For the sequencing of cellular clones, 1 *μ*g of purified PCR product was sequenced using same primer set as used in PCR amplification, and for the gene clones, replicate PCR products were pooled, separated on agarose gel, and band was purified using Gel extraction kit (GeNei, India). Clones were constructed with the Invitrogen TOPO TA cloning kit and purified using GeNei purification kit (GeNei, India). Purified plasmids were digested with *Eco*RI to separate inserts and run on agarose to determine insert size. The PCR products and plasmid insert were sequenced with the Big Dye terminator Cycle Sequencing kit (Applied Biosystems model no. 3130XL). Clone sequences were checked by CHIMERA-CHECK program (http://rdp.cme.msu.edu/). 

### 2.5. Phylogenetic Analysis

Scoring and data analysis of restriction profiles were done in a binary matrix, and the data was used for calculating Jaccard's similarity coefficient [27]. Dendrogram was constructed by neighbor-joining [28] unweighted pair group arithmetic mean (UPGMA) method [29]. In order to estimate the goodness of fit of cluster analysis, cophenetic value matrices were calculated and compared with the original similarity matrices that were UPGMA clustered and tree was constructed by NJ methods to classify unknown isolates into closely related standard isolates by using the NTSYSpc analysis package (Version 2.02e; Applied Biostatistics Inc., Setauket, New York, USA). Phylogenetic analysis was done according to what was previously described by Stach et al. [30]. Phylogenetic identity of actinomycetes was determined by RUDP-BLAST result, sequence was aligned by Clustal X and phylogeny calculations, and dendrogram was constructed by Mega 4.1 software package using maximum parsimony methods [31]. Bootstrap analysis was conducted using 1000 resampling of data.

### 2.6. Statistical Analysis

In order to compare the bacterial diversity within the samples, 16S rRNA gene sequences showing >97% sequence similarity were grouped into the same OTU (phenotype). The species diversity within a community or habitat is measured by alpha (*α*) diversity which has two components: species richness and evenness, and is calculated into single index, that is, Shannon and Wiener index. It was calculated by using software Ecosim (version 5.0.1) (http://www.ecosim.ca/ELCWebApp/ecological_land_classification/ELC_eTool.html). Rarefaction analysis was done using the site Online Calculation (http://fastgroup.sdsu.edu/cal_tools.htm). The 16S rRNA gene clone libraries were compared using principal component analysis (PCA). PCA was performed to group or separate samples based on the biogeochemical parameters (total count, pH values, SO_4_^2−^, Na^+^, K^+^, Ca^2+^ and Mg^2+^) and the percentage of OTUs in each sample. 

### 2.7. Nucleotide Sequence Accession Numbers

Gene bank nucleotide accession numbers for sequences and their respective identified names were mentioned in Supplementary table of the Supplementary Material available online at http://dx.doi.org/10.1155/2013/781301.

## 3. Results and Discussion

**Figure 2:**
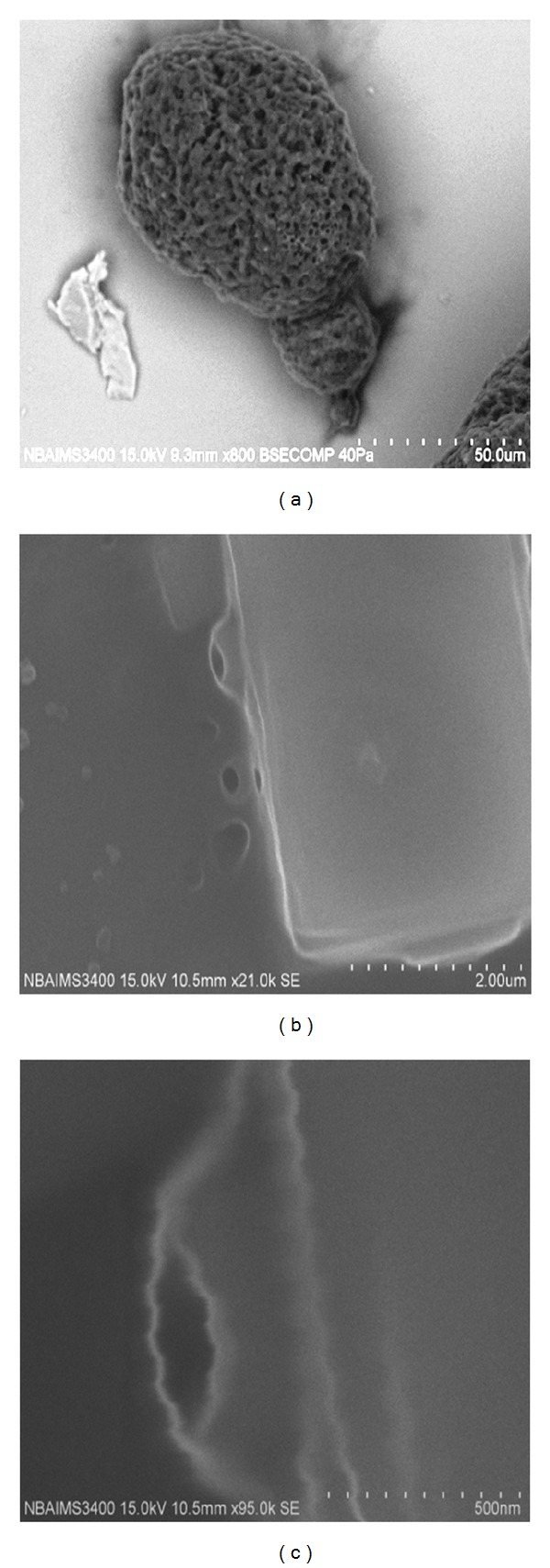
Scanning electron microscopy (SEM) of *Streptomyces* cellular isolate isolated from Sambhar salt lake, India, (a) showing globular sporangiophore, (b) sporangial pore, and (c) enlargement showing wavy sporangial pore architecture.

**Figure 3:**
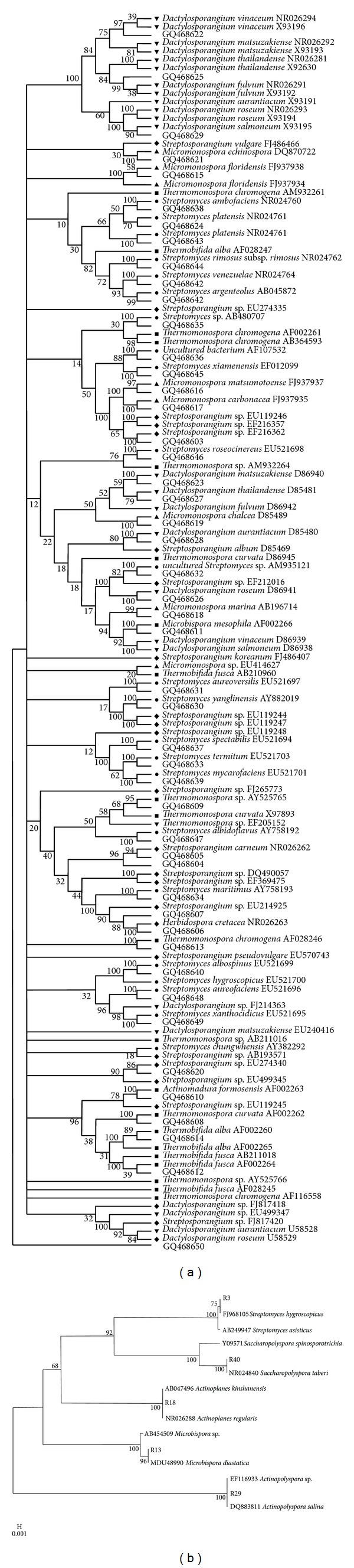
(a) Phylogenetic relationships among species of the genera *Streptomyces *(600 bp); *Micromonospora *(1 kb); *Streptosporangium *(500 bp); *Thermomonospora *(800 bp), and *Dactylosporangium *(580 bp) based on partial nucleotide sequences of the 16S rDNA. The tree was constructed using the maximum parsimony method. Percentages at nodes represent levels of bootstrap support from 1000 resampled datasets. GenBank accession numbers are given in parentheses and (b). Phylogenetic analysis of actinomycetes isolates from Sambhar salt lake based on partial nucleotide sequences (1.5 kb) of the 16S rDNA. The tree was constructed using the neighbor-joining method. Percentages at nodes represent levels of bootstrap support from 1000 resampled datasets. Bootstrap values less than 50% are not shown. The bar indicates 0.001% estimated sequence divergence

The aquatic regime for actinomycetes remains untouched for a long time in India [32], and species isolated from this biome are mostly novel with possible sources of extracellular enzymes and antibiotics [33]. Sambhar is the largest elliptical shaped salt lake which covers 3/4 salt productions in India. The actinomycetes density was found to vary between 1–13 × 10^4^ CFU mL^−1^ in the lake water samples. The cellular isolates were initially differentiated on the basis of microscopic (Figure 2), biochemical and chemotaxonomic parameters (Table 2). Further, based on 16S rDNA-RFLP fingerprinting all the isolates were clustered into five major groups, that is, I (7 isolates), II (17 isolates), III (6 isolates), IV (10 isolates), and V (6 isolates), and were identified primarily up to genera level as *Actinopolyspora* sp., *Streptomyces* sp., *Actinoplanes* sp., *Microbispora* sp., and *Saccharopolyspora* sp., respectively (Table 2). The representative isolates of each group were subjected to 16S rDNA sequencing, and postalignment followed by phylogenetic analysis was carried out with closest representative in RDP database and were identified as *Actinopolyspora salina* (R29), *Streptomyces hygroscopicus* (R3), *Actinoplanes regularis* (R18), *Saccharopolyspora taberi* (R40), and* Microbispora diastatica* (R13), respectively (Figure 3(b)).

 In uncultivable approaches, highest yield of DNA was recovered from surface water than other samples (surface water > 1.5 m > 2.5 m) respectively, followed by the amplification of 16S rDNA using specific primers of *Streptomyces*, *Micromonospora*, *Streptosporangium*, *Thermomonospora*, and *Dactylosporangium* of corresponding molecular weight of 0.6, 1.0, 0.5, 0.8, and 0.58 kb, respectively. Phylogenetic analysis of representative isolates sequences was done by multiple alignment of representative isolates with its closest relative sequences from public RDP database. A total of 409 clones were obtained from total samples of Sambhar salt Lake *Streptomyces* (32%) >  *Micromonosporaceae* (24%) >  *Streptosporangiaceae* (19%) >  *Dactylosporangiaceae* (14%) >  *Thermomonosporaceae* (11%) (Table 1). All the amplified PCR products of reference were further classified by RFLP analysis; the group representative clones were identified by sequencing. All clones were clustered together by Pattern Restriction Analysis fingerprint analysis; representative clones were sequenced and found to be a total of 55 OTUs by BLAST searches (Supplementary Table). One representative of each OTU was taken, and its sequence was aligned with its closest relatives. The analysis of the constructed phylogenetic tree (Figure 3(a)) revealed presence of clones branching among 5 actinobacterial divisions, including *Streptomyces* (130 clones, 20 OTUs), *Micromonospora *(96 clones, 7 OTUs), *Streptosporangium* (79 clones, 9 OTUs), *Thermomonospora* (46 clones, 8 OTUs), and *Dactylosporangium* (58 clones, 8 OTUs). Maximum number of clones was found from Surface lake water (133 clones) and minimum number from shallow water layer (77 clones). However, vertical profiling of actinomycetes population also showed that surface water had maximum number of clones (133), followed by lake water at the depth of 1.5 m (112) and at 2.5 m depth (87 clones) respectivelya and contained a majority of clones (OTUs) (Table 1). The Shannon and Wiener index in a Sambhar salt lake (on the basis of number of OTUs present in the different samples of lakes) was calculated to be 1.55, 1.52, 1.55, and 1.49 from surface lake water, lake water at depth of 1.5 m, shallow layer of water with algal population, and finally lake water at depth of 2.5 m, respectively. In this study we focused on cultivable and uncultivable diversity of actinomycetes at genus and species level as a first step toward a practical application of diversity analysis of these microbes. By using the classical approaches, we are able to identify 70% isolates up to generic level; it reduces isolate number that is required in the application of the more costly DNA sequence analysis. Molecular diversity study showed a good variability with 16S rDNA-RFLP of all the isolates with respect to their morphological and chemotaxonomic characteristics. 

 Restriction analysis has been used to study genotypic diversity analysis and differentiation of actinomycetes within genus and/or species levels [34–36]. Sambhar salt lake had good number of actinomycetes diversity, although culturable and unculturable diversity analysis techniques have their own advantages and disadvantages [37] by using polyphasic identification strategies for culture dependent actinomycetes, we identified 4 genera, of which *Streptomyces *(17 morphotypes) was a dominant genus followed by *Microbispora* (8), *Actinopolyspora* (7), *Saccharopolyspora* (6), and *Actinoplanes* (6), respectively, whereas for culture independent diversity, we found 52 species with 5 genera of actinomycetes, *namely*, *Streptomyces* followed by *Micromonospora*, *Streptosporangium*, *Dactylosporangium*, and *Thermomonospora*, respectively. Other studies also reported that *Streptomyces* is the dominant genus within actinobacterial population present from aquatic habitat [37] followed by *Micromonospora* [38]. Vijay et al. [39] reported 9 genera (*Streptomyces* as dominant genus) from 18 marine sediment samples (halophilic nature) from Bay of Bengal, India. The difference in diversity between various depths of extreme environments is clearly observed in Sambhar salt lake. Our result showed that surface water has total 133 clones with 47 OTUs followed by lake water at depth of 1.5 m (112 clones with 39 OTUs), lake water at depth of 2.5 m (87 clones with 39 OTUs). Field et al. [40] also reported that the bacterial community with respect to species composition in aquatic system decreases vertically from surface water. 

 The main cause of fluctuation of diversity may be attributed to nutrient scarcity and oxygen requirement at sediment than surface water [41]. Till date very less information regarding microbial diversity functions, such as nutrient cycling, degradation of xenobiotics, and ecosystem stability, is known. Application of diversity estimators coupled with diversity indices and community dominance enables the discrimination of environments, either geographically isolated or a particular specific niche. Extreme ecosystems that are characterized by high dominance of particular organisms require a smaller sampling size to determine the main elements of their community structure. Diversity indices were used to determine species richness and evenness into single index, that is, Shannon index, which indicates pattern of diversity, shifting of diversity, and so forth [42]. Our calculations also indicate that Shannon index decreases from surface water (1.55) to lake water at depth of 1.5 m (1.52,) and finally 2.5 m (1.49) at various depths of Sambhar lake, respectively. The implementation of above described techniques will enhance bioprospecting strategies in several respects like presence or absence of species in the environment being sampled; this information can be employed in the design of cultivation strategies. 

 Molecular characterization of all the isolates was carried out by restriction analysis of 16S rDNA with *Hha*I/*Cfo*I and *Taq*I restriction enzymes, respectively, and representative isolates were sequenced, while in the case of culture independent study, we used five specific primers for different *Actinomycetales* family followed by restriction analysis of amplified 16S rDNA products with same enzymes mentioned above and representative isolates were cloned, sequenced, and identified as *Streptomyces* as dominant genera in Sambhar salt lake by using both culture dependent and culture independent methods followed by *Microbispora* (culture dependent) and *Micromonospora* (culture independent techniques), respectively. Here we obtained evidence for presence of high species diversity of *Actinomycetes* in the Sambhar salt lake of India. The restriction enzymes used in present study specifically recognize the sequence “GCG/C” and “T/CGA.” The results of the different RFLP patterns obtained allowed us to effectively differentiate the strains into distinct groups of actinobacteria. This rapid and convenient method can be very useful in grouping actinobacterial isolates efficiently, although experimental caution must be taken during the phylogenetic analysis, while using the RFLP approach [43].

**Figure 4:**
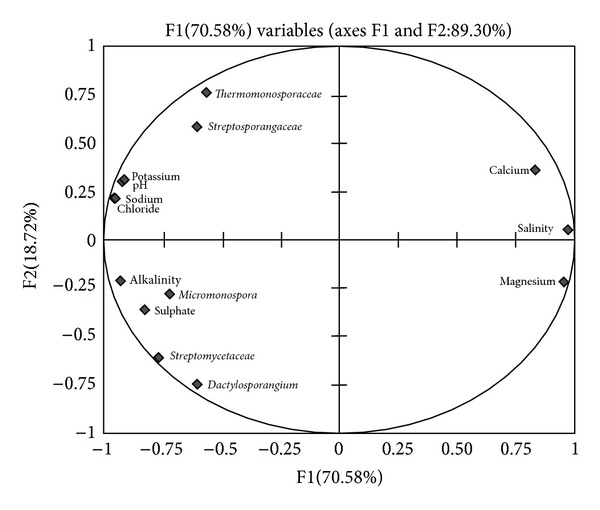
Rarefaction analysis showing sampling evenness. The inset graph shows reference ribotype plot.

 The result of PCA based on lake water properties are shown in Figure 4 and the principal component factor 1 and 2 explained 100% of the total variances. The PCA plot indicates that *Micromonosporaceae*, *Streptomycetaceae*, and *Dactylosporaceae* are closely grouped while their distribution was influenced by alkalinity and sulphate concentration while *Thermonosporaceae *and *Streptosporangaeae*  are grouped separately and their distribution is influenced by pH, potassium, sodium, and calcium ions concentration. Since biogeochemical properties may be influence by the heterogeneity observed in the samples with respect to specific OTUs. The Zhang et al. [44] demonstrated that microbial diversity was not related to Ca^2+^ concentration but features like pH influence the microbial community composition and diversity [45]. Thus it may be implicated that closely associated parameters and a key parameters that influence observed differences in the percentage of specific OTUs in the 16S rRNA gene libraries. Information about the composition of the microbial community is of key importance for better understanding of various processes in the aquatic ecosystem [46–48]. Molecular methods based on 16S rRNA are now widely used to gain insight into microbial communities [45, 49]. While environmental factors and the microbial community structure in various lakes have been well documented [47], the two aspects were often considered independently. 

**Figure 5:**
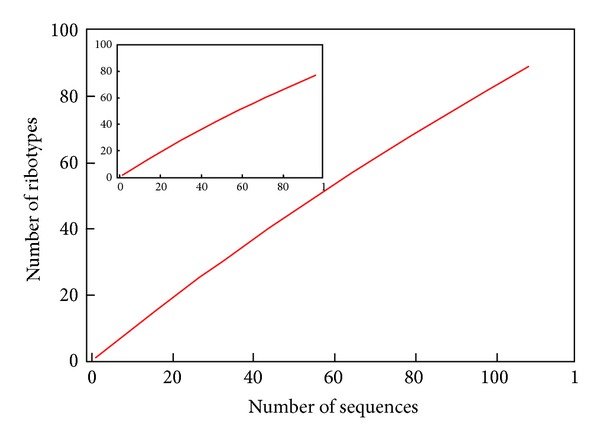
PCA analysis between the OTUs and biogeochemical parameters of Sambhar salt lake.

 The characterization of microbial community in different areas of a lake, mapped to differences in environmental parameters, may therefore provide information helpful in understanding the often complex processes. In recent years multivariate techniques such as principal component analysis (PCA) and others have been adopted to demonstrate the relationship between microbial community composition and environmental factors and have been proven to be more sensitive than univariate methods [50]. Using these techniques, the spatial and temporal variability in microbial community structure along with physiochemical factors, in lakes have been well documented, and several environmental parameters, such as nitrogen, pH, and so forth, have been considered to be the key factors driving the changes in community comparison [51, 52]. The rarefaction curves indicate that the actinobacterial population in the samples has a diversity coverage plateaued at 98.45 (Figure 5). The rarefaction curve analysis implied that these are likely to be minimum estimates of diversity parameters, such as Shannon index, Simpson's index, Coverage and evenness. Rarefaction curves having 98% confidence interval were constructed by comparing the number of clones in each 16S rRNA gene library. The coverage (C) of each 16S rRNA gene library, a measure of capture of diversity was calculated according to the equation *c* = 1 − (*n*/*N*) where n is the number of different OTU types from a clone library that were encountered only once and N is the total number of sequences of clones in the library [53]. Several statistical approaches can be used to analyze bacterial diversity estimates from the number of species found in relatively small samples [54]. Clone diversity was evaluated using Simpsons and Shannon-Wiener diversity indices. Both indices indicated a high diversity level of the microbial communities represented by the 16sRNA and cellular clones libraries. The Shannon-Wiener index is determined by OTU richness, whereas the Simpson index is highly influenced by the abundance of the most common OTU found in the sample [55] regarding the species richness. On the basis of the results of our investigation, we concluded that the actinomycetes diversity in the Sambhar salt lake is high at both genus and species level. So far, only a few reports were available of systematic investigation of actinomycetes diversity from salt lake of India, and these reports provide little or no information about the actinomycetes diversity at genus and species level. 

## 4. Conclusion

We examined 46 morphologically different actinomycetes isolates using the culture dependent isolates. They were grouped into five clusters on the basis of morphological, biochemical, and chemotaxonomic characteristics. Molecular characterization of all the isolates was carried out by restriction analysis of 16S rDNA with *Hha*I/*Cfa*I and *Taq*I restriction enzymes, respectively, and representative isolates were sequenced, while in the case of culture independent study, we used five specific primers for different *Actinomycetales* family followed by restriction analysis of amplified 16S rDNA products with same enzymes mentioned above, and representative isolates were cloned, sequenced, and identified as *Streptomyces* as dominant genera in Sambhar salt lake by using both culture dependent and culture independent methods followed by *Microbispora* (culture dependent) and *Micromonospora* (culture independent techniques), respectively. Here we obtained evidence for high species diversity of *actinomycetes* in the Sambhar salt lake of India. 

## Supplementary Material

Isolate names and accession numbers of representative isolates used in Clusta X alignment, Phylogeny calculations and dendrogram construction.

## Figures and Tables

**Table 1:** Cultural, biochemical, and chemotaxonomic characterization of cellular isolates.

Parameters	Actinomycetes strains used				
	R3	R29	R13	R40	R18
	Cell morphology (oat meal agar)				
Aerial mycelia colour	White	Grey	Yellow	White	Ash
Substrate mycelia colour	Yellow	Brown	Colorless	Yellow	Light brown
Soluble pigment	—	Brown	—	Yellow	—

	Growth pattern				
Optimum temperature	25–28	25–37	25–28	30–37	30–45
Optimum pH	7.0–8.0	7.0–7.5	6.5–7.5	7.0–8.0	7.0–7.8

	Degradation pattern				
Tween 80	*+ *	*+ *	*+ *	*+ *	*− *
Starch hydrolysis	*+ *	*+ *	*− *	*+ *	*+ *
Casein hydrolysis	*+ *	*+ *	*+ *	*+ *	*+ *
Tyrosine degradation	*+ *	*+ *	*+ *	*+ *	*− *
Xanthine degradation	*+ *	*+ *	*− *	*− *	*− *
Xylan	*− *	*+ *	*+ *	*+ *	*+ *
Urea	*− *	−	−	*+ *	*− *

	Carbon utilization pattern				
L-arabinose	−	*+ *	−	*+ *	*+ *
D-Fructose	*+ *	*+ *	−	*+ *	*+ *
D-Galactose	*+ *	*+ *	−	*+ *	*+ *
Glucose	*+ *	*+ *	*+ *	*+ *	*+ *
Sucrose	*+ *	*+ *	−	*+ *	*+ *
Starch	*+ *	*+ *	*+ *	*+ *	*+ *
D-Xylose	*− *	*+ *	*+ *	*+ *	*+ *
Rhamnose	*− *	*+ *	*+ *	*− *	*− *
Raffinose	*− *	*+ *	*+ *	*− *	*− *
Sodium acetate	*+ *	*+ *	*+ *	*− *	*+ *
Sodium citrate	*+ *	*− *	*− *	*− *	−
Sodium tartrate	*+ *	*− *	*− *	*+ *	*+ *

	Chemotaxonomy				
Cell wall type	IV	I	II	III	IV
Diaminopimelic acid	Meso	LL	Meso	Meso	Meso
Whole cell sugar	Arabinose, Galactose	Not characterized	Arabinose, Xylose	Glucose, Mannose, Ribose	Arabinose, Galactose

**Table 2:** Details of sample collected and frequencies of clones and OTU at different depths of Sambhar salt lake, India.

Samples	Sample type	% Salinity	No. of Clones/OTUs				
			(*Micromonosporace*)	(*Streptomycetaceae*)	(*Streptosporangaceae*)	(*Thermomonosporaceae*)	(*Dactylosporangiace*)
Surface lake water	Water	18	30/7	42/16	27/9	14/8	20/7
Lake water at depth of 1.5 m	Water	4	28/5	30/14	20/7	10/5	20/8
Shallow layer of water with algal population	Water	25	18/6	24/10	15/5	10/4	10/6
Lake water at depth of 2.5 m	Water	7	20/4	30/15	17/8	12/7	8/5

Total clones picked	—	—	96	130	79	46	58
