# Supplementary material for: Actinomycetes Diversity among rRNA Gene Clones and Cellular Isolates from Sambhar Salt Lake, India
Source: ScientificWorldJournal. 2013 Nov 6;2013:781301. doi: 10.1155/2013/781301 (PMC3836413; doi:10.1155/2013/781301)
Supplement: Supplementary file 1 — Isolate names and accession numbers of representative isolates used in Clusta X alignment, Phylogeny calculations and dendrogram construction. [file 781301.f1.docx]

**Supplementary Table:** Isolate names and accession numbers of representative isolates.

| **S. No** | **Representative isolates/ clones** | **Species** | **Accession numbers** |
| --- | --- | --- | --- |
|  | R13 | *Microbispora diastatica* | GQ390360 |
|  | R29 | *Actinopolyspora salina* | GQ390362 |
|  | R40 | *Saccharopolyspora taberi* | GQ390363 |
|  | R3 | *Streptomyces hygroscopicus* | GQ390361 |
|  | R18 | *Actinoplanes regularis* | GQ390364 |
|  | clone 219 | *uncultured Dactylosporangium salmoneum* | GQ468629 |
|  | clone 216 | *uncultured Dactylosporangium aurantiacum* | GQ468628 |
|  | clone 215 | *uncultured Dactylosporangium thailandense* | GQ468627 |
|  | clone 211 | *uncultured Dactylosporangium roseum* | GQ468626 |
|  | clone 204 | *uncultured Dactylosporangium fulvum* | GQ468625 |
|  | clone 217 | *uncultured Dactylosporangium sp,* | GQ468624 |
|  | clone 212 | *uncultured Dactylosporangium matsuzakiense* | GQ468623 |
|  | clone 201 | *uncultured Dactylosporangium vinaceum* | GQ468613 |
|  | clone 180 | *uncultured Thermomonospora curvata* | GQ468608 |
|  | clone 167 | *uncultured Thermomonospora sp.* | GQ468609 |
|  | clone 163 | *Thermomonospora formosensis* | GQ468610 |
|  | clone 177 | *uncultured Microbispora mesophila* | GQ468611 |
|  | clone 178 | *uncultured Thermomonospora fusca* | GQ468612 |
|  | clone 175 | *uncultured Thermomonospora chromogena* | GQ468613 |
|  | clone 182 | *uncultured Thermomonospora alba* | GQ468614 |
|  | clone 61 | *uncultured Streptosporangium spp.* | GQ411540 |
|  | clone 62 | *uncultured Streptosporangium album* | GQ411539 |
|  | clone 63 | *uncultured Streptosporangium koreanum* | GQ411542 |
|  | clone 64 | *uncultured Streptosporangium pseudovulgare* | GQ411541 |
|  | clone 71 | *uncultured Streptosporangium vulgare* | GQ468603 |
|  | clone 68 | *Streptosporangium claviforme* | GQ468604 |
|  | clone 69 | *uncultured Streptosporangium carneum* | GQ468605 |
|  | clone 68 | *uncultured Streptosporangium corrugatum* | GQ468606 |
|  | clone 67 | *uncultured Streptosporangium longisporum* | GQ468607 |
|  | clone 1 | *uncultured Micromonospora echinospora* | GQ468621 |
|  | clone 14 | *uncultured Micromonospora sp* | GQ468620 |
|  | clone 2 | *uncultured Micromonospora floridensis* | GQ468615 |
|  | clone 48 | *uncultured Micromonospora chalcea* | GQ 468619 |
|  | clone 45 | *uncultured Micromonospora marina* | GQ 468618 |
|  | clone 4 | *uncultured Micromonospora carbonacea* | GQ 468617 |
|  | clone 3 | *uncultured Micromonospora matsumotoense* | GQ 468616 |
|  | clone 329 | *uncultured Streptomyces yanglinensis* | GQ 468630 |
|  | clone 335 | *uncultured Streptomyces aureoversilis* | GQ 468631 |
|  | clone 324 | *uncultured Streptomyces sp.* | GQ 468632 |
|  | clone 330 | *uncultured Streptomyces termitum* | GQ 468633 |
|  | clone 340 | *uncultured Streptomyces maritimus* | GQ 468634 |
|  | clone 325 | *uncultured Streptomyces sp.* | GQ 468635 |
|  | clone 342 | *uncultured bacterium* | GQ 468636 |
|  | clone 338 | *uncultured Streptomyces spectabilis* | GQ 468637 |
|  | clone 320 | *uncultured Streptomyces albofaciens* | GQ 468638 |
|  | clone 331 | *uncultured Streptomyces mycarofaciens* | GQ 468639 |
|  | clone 333 | *uncultured Streptomyces albospinus* | GQ468640 |
|  | clone 323 | *uncultured Streptomyces venezuelae* | GQ468641 |
|  | clone 327 | *uncultured Streptomyces argenteolus* | GQ468642 |
|  | clone 321 | *uncultured Streptomyces platensis* | GQ468643 |
|  | clone 326 | *uncultured Streptomyces rimosus* | GQ468645 |
|  | clone 328 | *uncultured Streptomyces xiamenensis* | GQ468645 |
|  | clone 334 | *uncultured Streptomyces roseocinereus* | GQ468646 |
|  | clone 339 | *uncultured Streptomyces hygroscopicus* | GQ468647 |
|  | clone 336 | *uncultured Streptomyces aureofaciens* | GQ468648 |
|  | clone 337 | *uncultured Streptomyces xanthocidicus* | GQ468649 |
|  | clone 341 | *uncultured Streptomyce chungwhensiss* | GQ468650 |
